# Metastatic squamous cell carcinoma of unknown primary: a case report and brief literature review

**DOI:** 10.3389/fonc.2025.1613500

**Published:** 2025-10-24

**Authors:** Ying Yang, Wei Sun, Jun Jia, Jing Yu, Zhiwei Sun, Feng Du, Youwu Shi, Jing Sun, Songlin Gao, Yanjie Xiao, Xiaodong Zhang

**Affiliations:** ^1^ Key Laboratory of Carcinogenesis and Translational Research (Ministry of Education, China), The VIP-II Gastrointestinal Cancer Division of Medical Department, Peking University Cancer Hospital and Institute, Beijing, China; ^2^ Key Laboratory of Carcinogenesis and Translational Research (Ministry of Education), Department of Pathology, Peking University Cancer Hospital and Institute, Beijing, China

**Keywords:** carcinoma of unknown primary, squamous cell carcinoma, multimodal therapy, comprehensive workup, molecular testing

## Abstract

Carcinoma of unknown primary (CUP) is a rare malignancy characterized by metastatic disease without an identifiable primary tumor, even after extensive diagnostic evaluation. This case report described a 70-year-old female patient with squamous cell CUP (SCCUP) who initially presented with elevated carbohydrate antigen 19–9 and a diaphragmatic mass. Despite comprehensive workup, including ^18^F-fluorodeoxyglucose positron emission tomography–computed tomography and a 90-gene expression assay, the primary site remained unclear. The patient underwent surgical resection followed by two cycles of systematic therapy and achieved a disease-free survival of 14 months. This case underscores the limitations of the current diagnostic tools and the potential role of multimodal therapy in the management of CUP. The discordance between molecular testing and the clinical findings further emphasizes the perplexing nature of CUP. This report also reviews the literature on diagnosis and therapeutic options. Due to the absence of standardized regimens, future international collaboration and comprehensive genomic profiling are warranted to advance the understanding of this heterogeneous disease.

## Introduction

Carcinoma of unknown primary (CUP) presents only metastatic cancers without an identifiable primary tumor site, even after thorough clinical evaluations and various tests. Primary lesions have been identified only in less than 30% of patients despite comprehensive examinations (1, 2). Currently, the detection rate is still as low as 50% even diagnosed by positron emission tomography–computed tomography (PET-CT) and biopsy (3–5). It is assumed that the primary tumor of CUP is below the minimum detectable lesion by current techniques, which may be due to the natural disease regression. The reported incidence of CUP ranges from 2.3% to 5% (6–8), with a median age at diagnosis of 65 years and a slight male predominance (8, 9). The median overall survival (OS) is 6–10 months (7, 10), while the 5-year survival is 5%–15% (11). Adenocarcinoma accounts for 40%–60% of CUP cases, and squamous cell carcinoma represents 15%–20% (10, 11). Here, we present a case of CUP diagnosed and treated by our cancer division.

The patient provided written informed consent for the publication of this case report, including all associated clinical details and images. All identifying information has been anonymized to protect the patient’s privacy.

## Case description

### General information

The patient was a 70-year-old Chinese Han woman with an Eastern Cooperative Oncology Group Performance Status (ECOG PS) of 1. She was 155 cm in height and 63 kg in weight and with a body surface area of 1.62 m^2^. Her blood group is Rh-negative type B. The patient did not report any obvious discomfort, and physical examinations revealed no remarkable findings. She had a medical history of hypertension that was well controlled by oral valsartan/hydrochlorothiazide combination therapy. She denied any history of tobacco or alcohol use, as well as any family history of malignancy.

### Diagnostic workup

**Figure 1:**
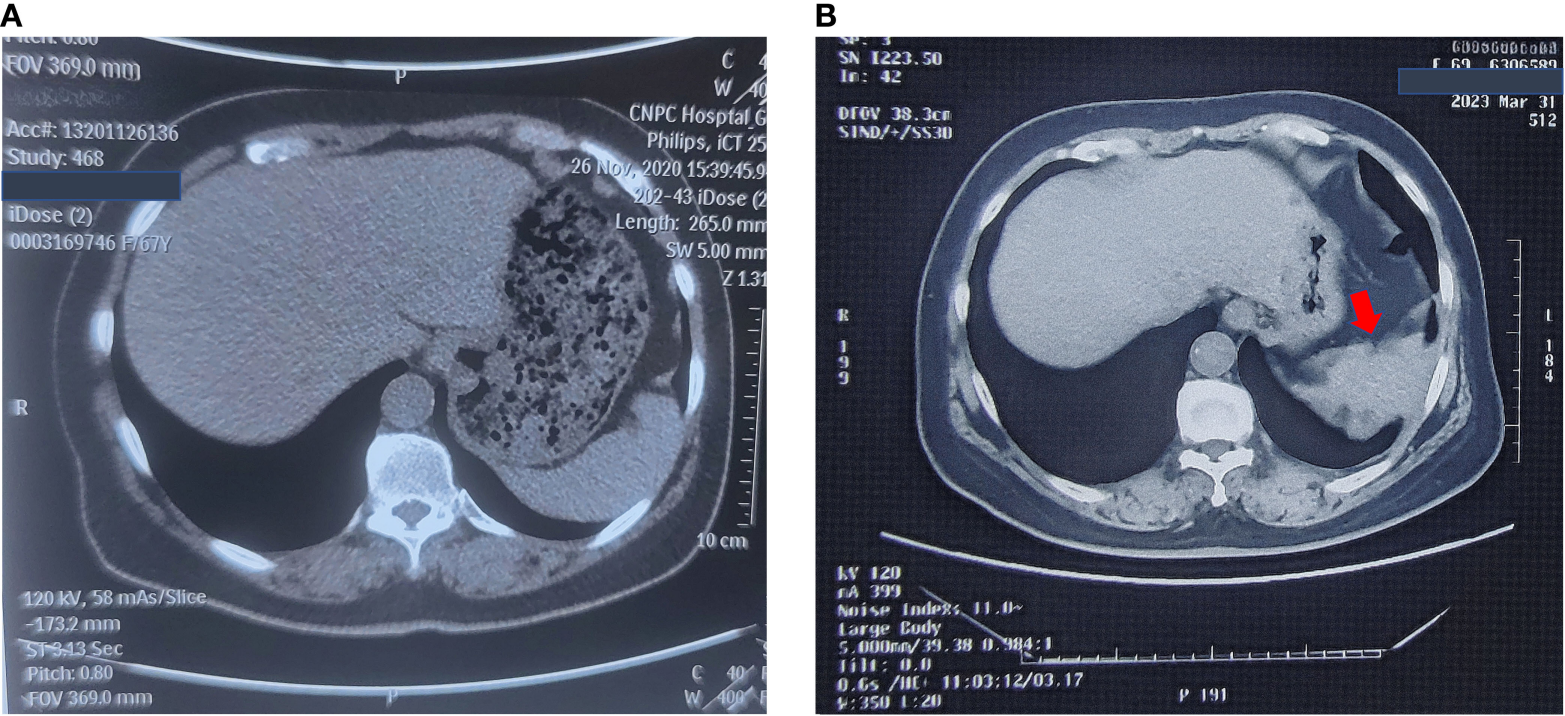
No-contrast abdomen CT changes before November, 2023. **(A)** No-contrast abdomen CT on November 26^th^, 2020. **(B)** No-contrast abdomen CT on March 31^st^, 2023.

Carbohydrate antigen 19-9 (CA19-9) was incidentally found to be elevated, i.e., 56 U/ml (normal range = 0–37 U/ml), in October 2020, but no abnormal imaging findings were detected in November. Her CA19–9 had been progressively increasing since then. In May 2022, a colonoscopy showed no abnormalities. In March 2023, non-contrast abdominal CT revealed a space-occupying lesion near the spleen in the subphrenic region, but was not given attention for further investigation. The CT imaging changes are shown in **Figure 1**. In April 2023, gastroscopy identified a 2 cm × 1.5 cm pedunculated polyp near the cardia of the gastric greater curvature. This was pathologically confirmed as a hyperplastic polyp.

**Figure 2:**
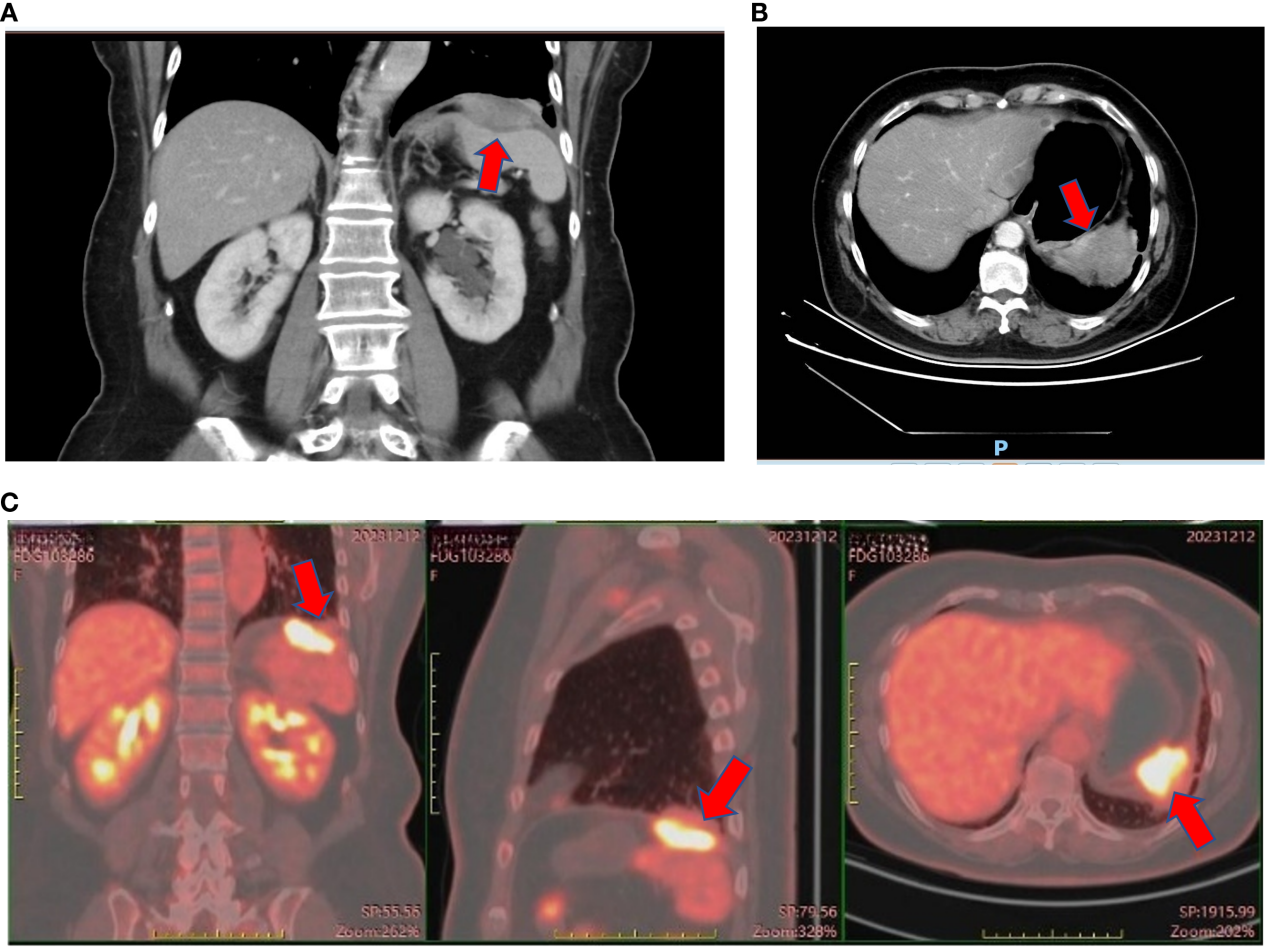
Enhanced CT and PET-CT of the left subdiaphragmatic mass. **(A)** Coronal view of enhanced CT of the left subdiaphragmatic mass on November 14^th^, 2023. **(B)** Axial view of enhanced CT of the left subdiaphragmatic mass on November 14^th^, 2023. **(C)** PET-CT of the left subdiaphragmatic mass on December 12^th^, 2023.

On November 13, 2023, her carcinoembryonic antigen (CEA) level was 43.52 ng/ml (normal range = 0–5 ng/ml), CA19–9 was 387 U/ml, and neuron-specific enolase (NSE) was 17.3 ng/ml (normal range = 0–15.2 ng/ml). Enhanced abdominal and pelvic CT revealed post-polypectomy changes in the stomach, with no obvious wall thickening or abnormal enhancement of the gastric wall. A spindle-shaped lesion with soft tissue density was observed below the left diaphragm, measuring approximately 5 cm × 1.5 cm. The lesion exhibited heterogeneous enhancement, smeared-out boundaries, and indistinct separation from the diaphragm. The spleen was shoved. The lesion was highly suspicious for a mesenchymal tumor, although malignant metastasis could not be ruled out. The left pleura appeared slightly thickened, and a patchy consolidation was noted adjacent to the pleura in the lower lobe of the left lung. On December 11, 2023, ^18^F-fluorodeoxyglucose (FDG) PET-CT revealed an irregular soft tissue mass in the left diaphragmatic area with a maximum standardized uptake value (SUV_max_) of 11.4, which measured approximately 3.9 cm × 3.4 cm × 2.4 cm. The lesion was growing toward the diaphragm; and its boundaries with the diaphragm and the spleen were unclear. PET-CT also demonstrated local thickening of the adjacent left pleura with a mildly increased radiotracer uptake (SUV_max_ = 2.3), as well as local discoid atelectasis of the lung. No enlarged or hypermetabolic lymph nodes were observed in the retroperitoneum or abdominal cavity. The lesion was considered suspicious for a mesenchymal tumor. The CT and PET-CT images are shown in **Figure 2**. However, the patient did not undergo a biopsy due to her Rh-negative blood type.

### Treatment

On January 5, 2024, the patient underwent resection of the diaphragmatic mass and the spleen. The pathology findings were as follows:

1. The spleen measured 8.5 cm × 6 cm × 4 cm and the diaphragm 9.5 cm × 8 cm × 2 cm. A mass was observed within the diaphragm, measuring 6 cm × 5 cm × 2 cm. The cut section was grayish white and grayish yellow, solid, firm, and poorly demarcated from the surroundings. The focal lesion was in close proximity to the spleen.
2. The squamous cell carcinoma was moderately differentiated.
3. The tumor did not involve the spleen parenchyma, and no tumor was observed at the diaphragmatic resection margin.
4. The immunohistochemical results showed P16 (mixed +), P40 (+), CK5/6 (cytokeratin 5/6) (+), calretinin (−), D2-40 (focal +), WT1 (Wilms tumor protein 1) (−), EBER (Epstein–Barr virus-encoded small ribonucleic acid, RNA) (−), and PD-L1(22C3) [programmed death-ligand 1 (22C3 clone)] combined positive score (CPS) of 40.

**Figure 3:**
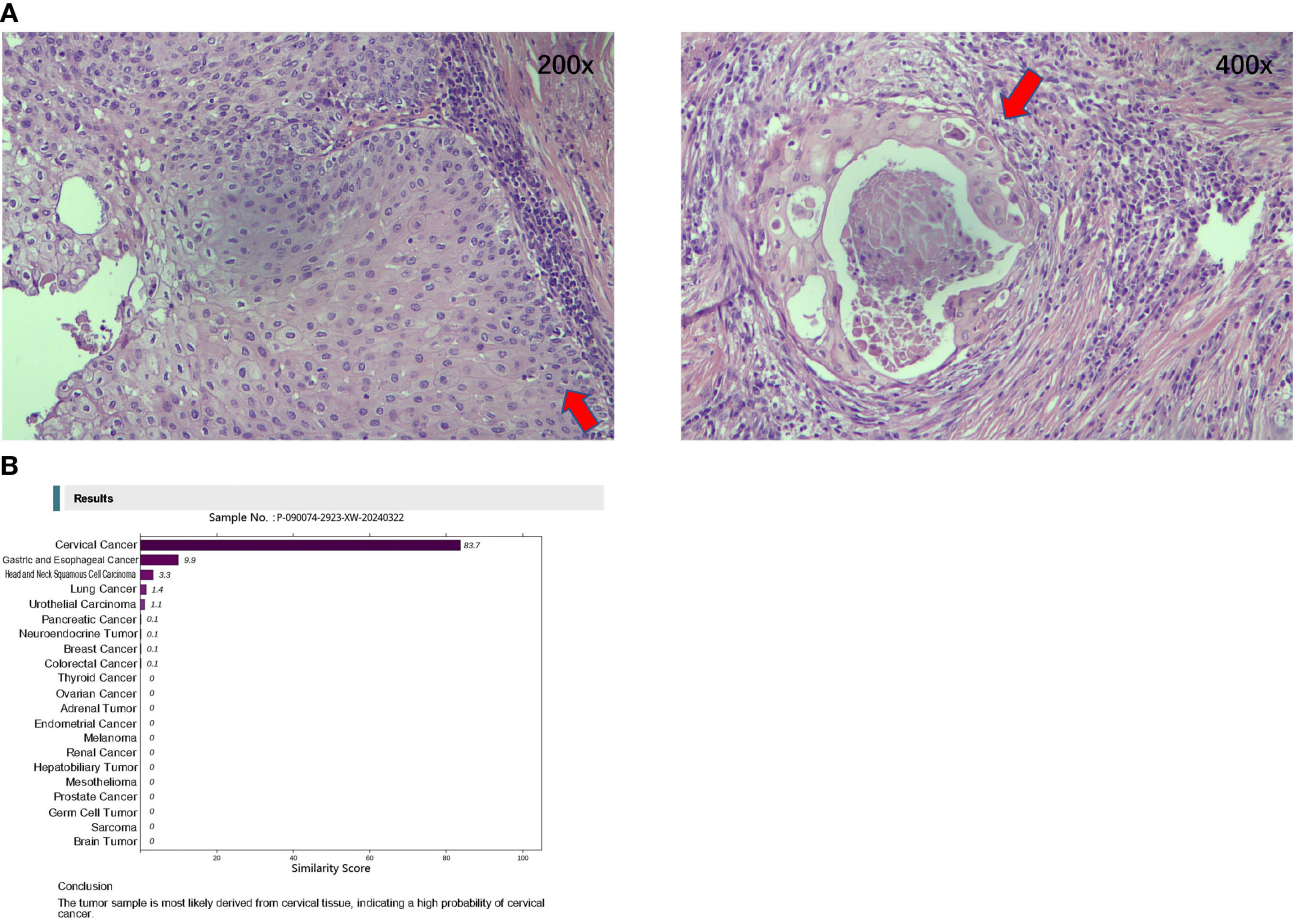
Hematoxylin and Eosin-stained pathological diagnosis and the Canhelp^®^-Origin 90-gene expression assay of resected lesions. **(A)** Hematoxylin and Eosin stained pathological diagnosis of resected lesions (Left: 200x; Right: 400x). **(B)** Canhelp^®^-Origin 90-gene expression assay of resected lesions.

The pathology images are shown in **Figure 3**. The pathologist recommended a thorough examination to rule out metastasis. A postoperative follow-up on March 1, 2024, showed a CEA of 1.38 ng/ml, CA19-9 of 10.1 U/ml, and NSE of 18.0 ng/ml.

From March 14 to April 7, 2024, two cycles of postoperative systematic therapy were administered. The regimen included albumin-bound paclitaxel (300 mg on day 1), carboplatin (500 mg on day 1), and pembrolizumab (200 mg on day 1), administered every 21 days. The main side effect was a grade 2 rash. After two cycles, the patient refused to continue.

### CUP workup

On March 26, 2024, a 90-gene expression assay (Canhelp^®^-Origin; Canhelp^®^ Genomics, Hangzhou, China) for CUP was conducted to identify the suspected origin. The results showed that the possible origin was the cervix uteri. The molecular analysis for origin is shown in **Figure 3**.

**Figure 4:**
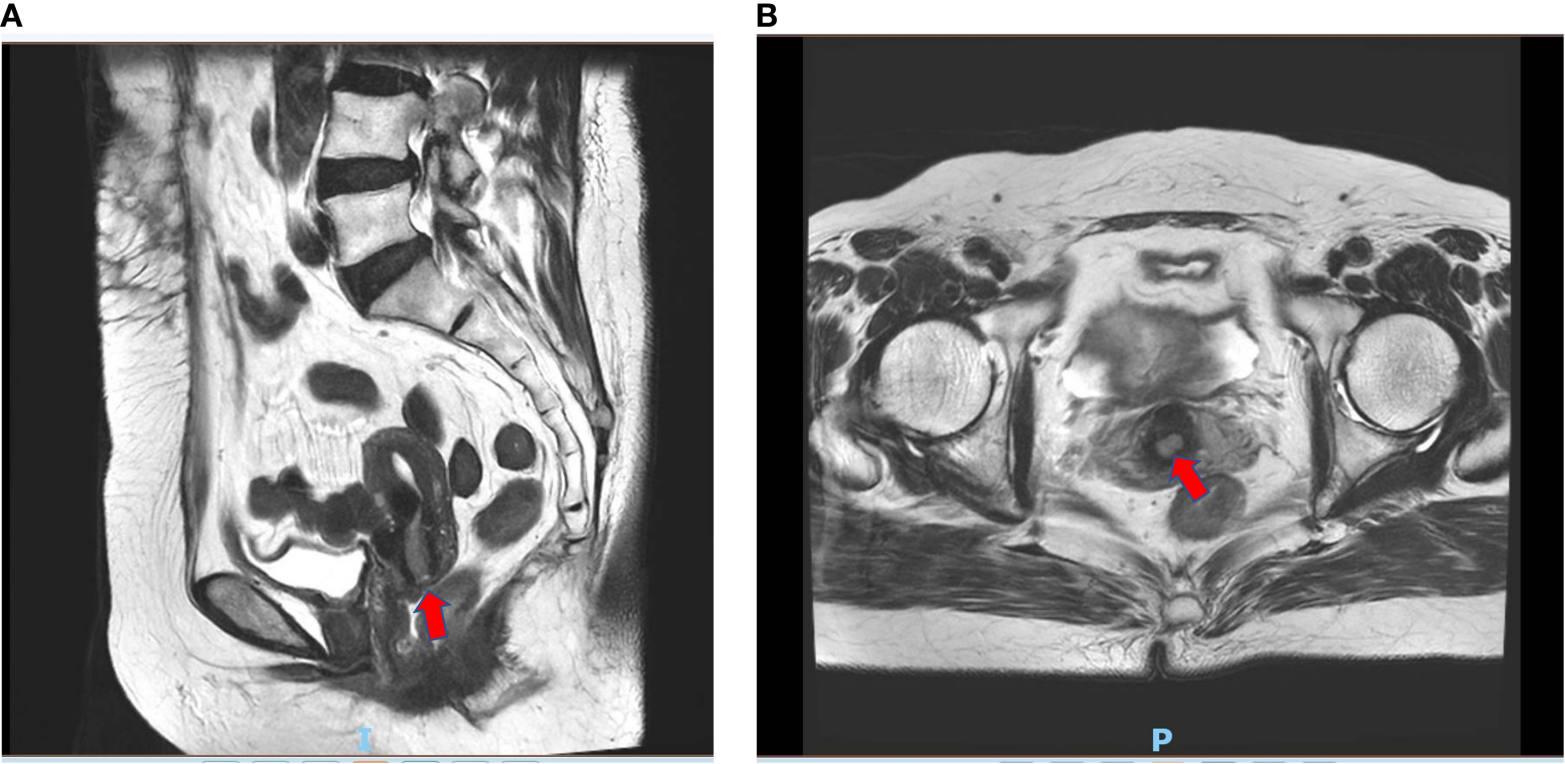
Enhanced magnetic resonance imaging of suspected cervical lesion on April 20th 2024. **(A)** Sagittal view of enhanced magnetic resonance imaging of suspected cervical lesion on April 20^th^ 2024. **(B)** Axial view of enhanced magnetic resonance imaging of suspected cervical lesion on April 20^th^ 2024.

Pelvic enhanced magnetic resonance imaging showed a thickened mucous membrane of the cervical canal, which was a patchy long T1 and long T2 signal lesion and measured approximately 12 mm × 8 mm × 18 mm. The lesion exhibited a high signal on diffusion-weighted imaging (DWI) and a strong enhancement, with a continuous low signal at the local cervical junctional zone. The images are presented in **Figure 4**. However, on April 3, 2024, a gynecologic examination and colposcopy did not find any abnormalities. The cervical pathology displayed in **Supplementary Figure S1** suggested atrophic signs, and the high-risk human papillomavirus (HPV) subtypes were all negative.

### Follow-up

On November 28, 2024, carbohydrate antigen 724 (CA724) was found to be elevated at 1,502 U/ml (normal range = 0–6.7 U/ml) during routine follow-up, but there were no abnormal signs on the CT scan. Compared with the PET-CT on December 11, 2023, the most recent imaging did not find any recurrent or metastatic features. The size of the bilateral hilar lymph nodes remained unchanged from previous imaging, with newly noted mild uptake increase (SUV_max_ = 4.0), which was suggestive of an inflammatory change, as seen in **Supplementary Figure S2**. On December 5, 2024, the CA724 level decreased to 203 U/ml. The changes in the tumor markers during the entire clinical course are shown in **Supplementary Figure S3**.

Up to April 17, 2025, when this manuscript was submitted, the disease-free survival (DFS) had been 15 months.

The timeline with relevant data and events is shown in **Supplementary Table S1**.

## Discussion

We presented a case of squamous cell CUP (SCCUP). The 70-year-old female patient underwent surgery and remained without evidence of disease for more than 15 months. The 90-gene expression assay (Canhelp^®^-Origin) suggested a primary origin in the cervix uteri. However, colposcopy and its corresponding pathology findings and the HPV test were negative.

A systematic review on 24 reported SCCUP cases in the pelvic, abdominal, and retroperitoneal regions demonstrated a median age at diagnosis of 56.5 years (range = 27–78 years), with a female-to-male ratio of 3:1, and HPV infection was confirmed in 2 out of 10 patients tested (12). The optimal treatment strategy for this designated SCCUP remains debatable. Cisplatin-based chemoradiation has been the most widely employed, and surgical resection may be considered for bulky disease (12). Another study indicated that localized abdominal SCCUP may predict more favorable outcomes compared with other CUP subgroups (13). The case presented here shares similar features to those of previous reports.

Two cycles of systematic therapy combining albumin-bound paclitaxel, carboplatin, and pembrolizumab were administered after surgery, but was discontinued due to patient refusal, consistent with the limited evidence for postoperative chemotherapy in CUP. The majority of previous studies have explored chemotherapy as a palliative treatment, and no definitive regimen has been established. Briasoulis et al. (14) reported a response rate of carboplatin plus paclitaxel of 38.7% [95% confidence interval (CI) = 27.5–49.9] and a median OS of 13.0 months. A small-sample study conducted by Nishimori et al. (15) reported that cisplatin plus docetaxel showed a response rate of 62.5%, with a median OS of 22.7 months. A meta-analysis conducted by Lee et al. (16) reported a median OS for CUP of 9.0 months (95%CI = 8.1–9.8) and demonstrated an improved survival trend with platinum- or taxane-based regimens compared with other chemotherapy regimens. However, this association did not reach statistical significance with prolonged follow-up and after multivariate adjustment. A multidisciplinary treatment that integrated palliative surgery, radiotherapy, and chemotherapy suggests a possible survival benefit (17). Our case exhibited better outcomes than those reported in the meta-analysis. This good prognosis may further verify the suspected primary of cervical cancer that showed a less aggressive biological behavior. It could also reflect the effectiveness of the comprehensive treatment strategy comprising surgery, chemotherapy, and immunotherapy. In addition, in patients administered carboplatin plus paclitaxel therapy, the researchers observed that those with a PS of 0–1 and no bone metastasis had significantly better survival compared with patients with a PS of ≥2 or with bone metastasis (1-year OS = 67.1% *vs*. 36.8%, *p* = 0.0003) (18). Our patient had a PS of 1, with no evidence of bone or visceral metastasis, and underwent radical surgery. These factors may have also contributed to the better survival outcome.

Canhelp^®^-Origin is a 90-gene expression profiling assay created for differential diagnosis among 21 prevalent malignancies. These tumor specimens include breast, lung, colorectal, prostate, ovarian, and pancreatic carcinomas, as well as adrenal, renal, thyroid, hepatic, endometrial, cervical, gastroesophageal, and neuroendocrine lesions. In addition, the assay detects melanomas, mesotheliomas, sarcomas, germ cell tumors, and malignancies of the head and neck region and the urinary tract. This methodology employs reverse transcription polymerase chain reaction analysis, utilizing total RNA obtained from formalin-fixed, paraffin-embedded tumor tissues (19). The performance of the 90-gene assay has been verified, demonstrating a sensitivity of 95.7% and a specificity of 99.0% for cervical cancer. The accuracy for squamous cell carcinoma is 91.0%, which is slightly lower than that for adenocarcinoma (95.2%) (20). Despite the high sensitivity, specificity, and accuracy of the test, no malignant lesions were identified in the patient’s cervix. This provides further proof of the challenge of identifying the origin of CUP and may suggest that the primary lesion has regressed during the disease course. Several mechanisms have been proposed for the regression. For example, early metastasis occurs before the primary tumor is clinically detectable, followed by immune-mediated elimination of the primary lesion, while metastatic clones continue to grow. Furthermore, the metastatic cells may acquire a pro-metastatic phenotype with genetic or epigenetic changes and thrive independently of the primary tumor. Moreover, modern techniques may fail to detect a tiny primary tumor (21).

Another advantage of the 90-gene expression assay is that it guides site-specific therapy. In the Fudan CUP-001 study, 91 of the 182 patients received site-specific therapy based on the results of the 90-gene expression assay, among whom 45% were administered targeted agents or immunotherapy, such as bevacizumab, PD-1 inhibitors, and epidermal growth factor receptor tyrosine kinase inhibitors (EGFR-TKIs). The other 91 patients received empirical chemotherapy, and only 26% were administered targeted agents or immunotherapy. The median progression-free survival (PFS) was 9.6 months, while the median OS was 28.2 months for patients who received site-specific therapy *versus* the corresponding 6.6 and 19.0 months for those who received empirical chemotherapy [PFS: unadjusted hazard ratio (HR) = 0.68, 95%CI = 0.49–0.93, *p*=0.017; OS: unadjusted HR = 0.74, 95%CI = 0.52–1.06, *p*=0.098]. Notably, among the 40 patients with SCCUP, site-specific therapy significantly improved the PFS compared with empirical chemotherapy (median PFS = 17.2 *versus* 4.7 months, HR = 0.41, *p*=0.017) (22). In our case, the patient received two cycles of chemotherapy combined with immunotherapy following surgery. However, given the postoperative setting and the limited number of treatment cycles, any definitive conclusions should be drawn with caution.

The major limitations of CUP, in particular SCCUP, include the lack of large-scale clinical studies and biological experiments elucidating the underlying molecular mechanisms. A recent large-scale comprehensive genomic profiling (CGP) study of 443 cases of SCCUP has revealed unique molecular characteristics distinct from those of non-squamous CUP, including actionable biomarkers (23). The integration of CGP into diagnostic workflows could improve the management of this challenging malignancy, which is a potential future direction. Given the rarity of this disease, multicenter collaboration, potentially on an international scale, is essential to accumulate sufficient cases in order to explore the nature of CUP.

## Data Availability

The original contributions presented in the study are included in the article/**Supplementary Material**. Further inquiries can be directed to the corresponding author.

## Supplementary material

Supplementary Figure 1 Cervical pathology (40x).

Supplementary Figure 2 Follow-up PET-CT on November 29^th^, 2024.

Supplementary Figure 3 Tumor marker change trends during the disease course. **(A)** CA19-9 change trends during the disease course. **(B)** CEA change trends during the disease course. **(C)** CA724 change trends during the disease course. **(D)** NSE change trends during the disease course.
