# Supplementary figures and images for: Metastatic squamous cell carcinoma of unknown primary: a case report and brief literature review
Source: Front Oncol. 2025 Oct 24;15:1613500. doi: 10.3389/fonc.2025.1613500 (PMC12591943; doi:10.3389/fonc.2025.1613500)

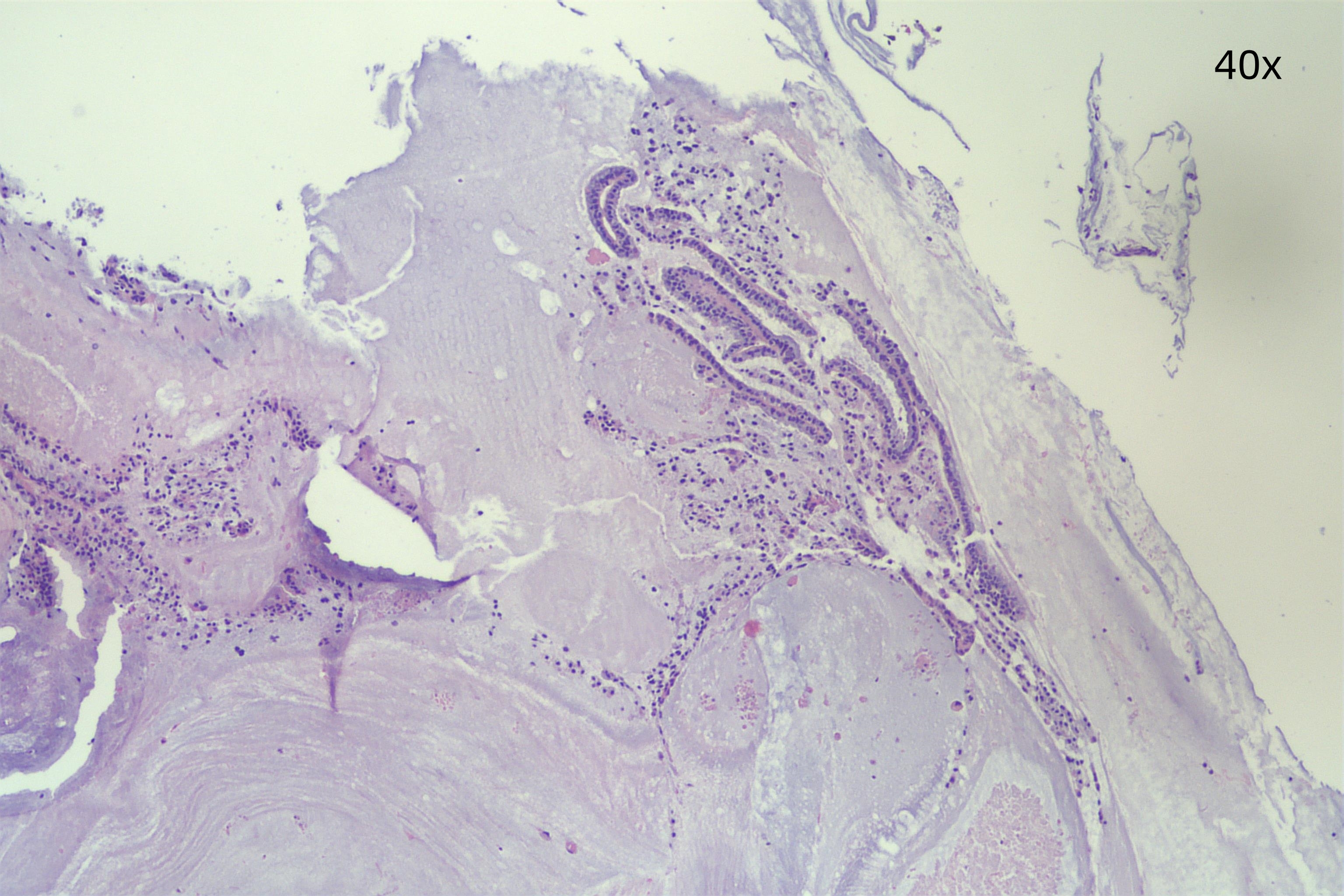

Supplement: Supplementary Figure 1 — Cervical pathology (40x). [file Image1.jpeg]

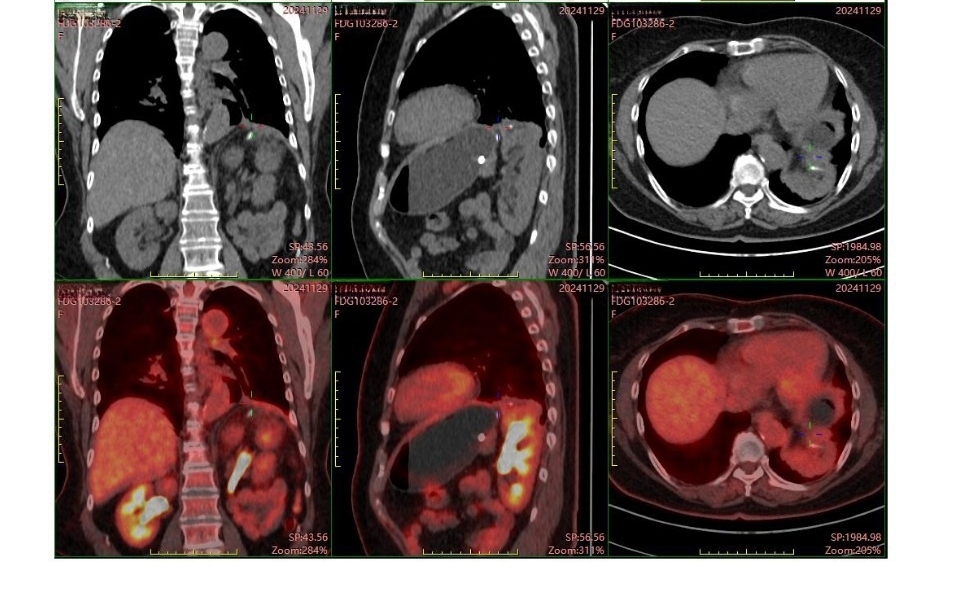

Supplement: Supplementary Figure 2 — Follow-up PET-CT on November 29th, 2024. [file Image2.jpeg]

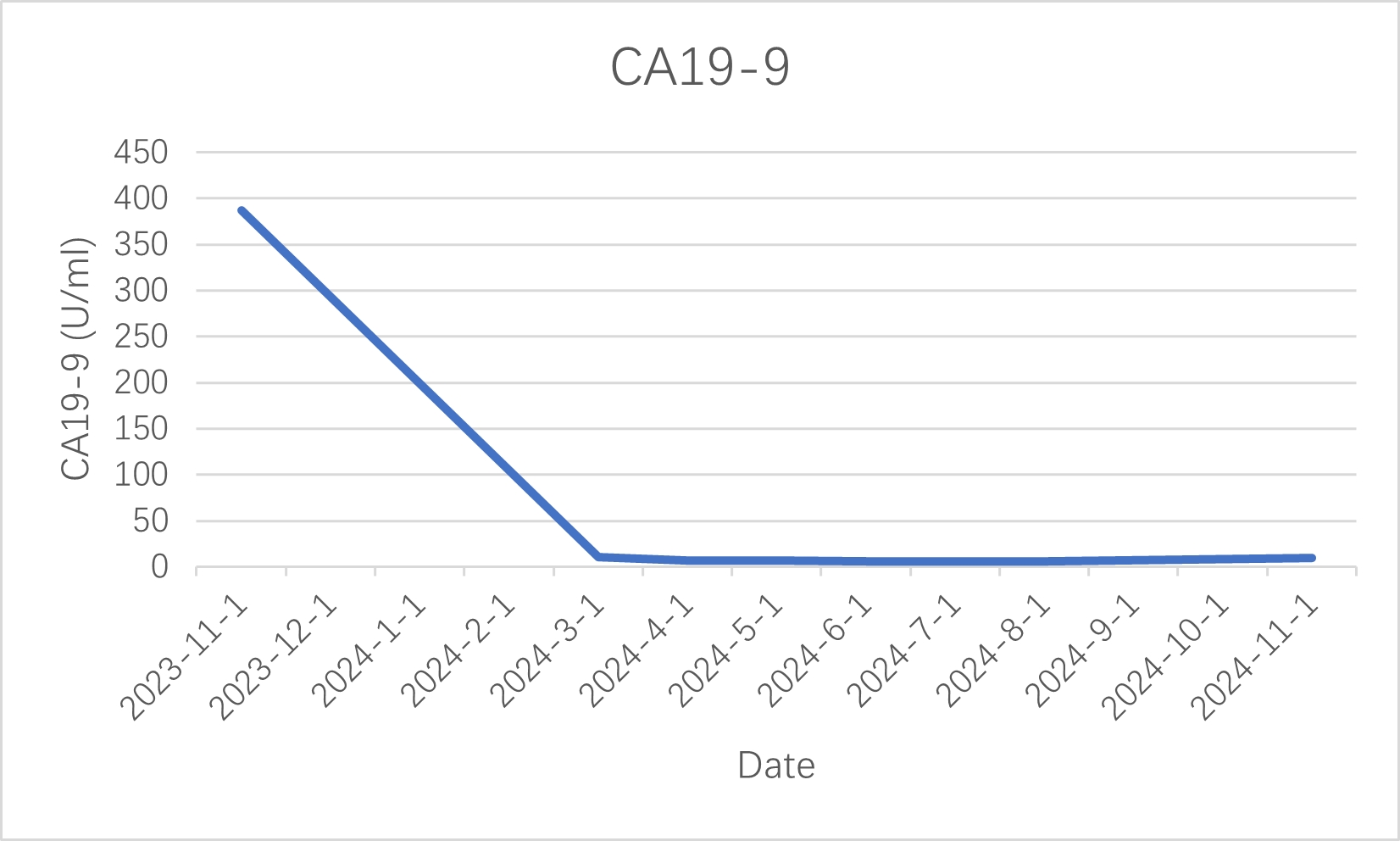

Supplement: Supplementary Figure 3 — Tumor marker change trends during the disease course. (A) CA19-9 change trends during the disease course. (B) CEA change trends during the disease course. (C) CA724 change trends during the disease course. (D) NSE change trends during the disease course. [file Image3.jpeg]

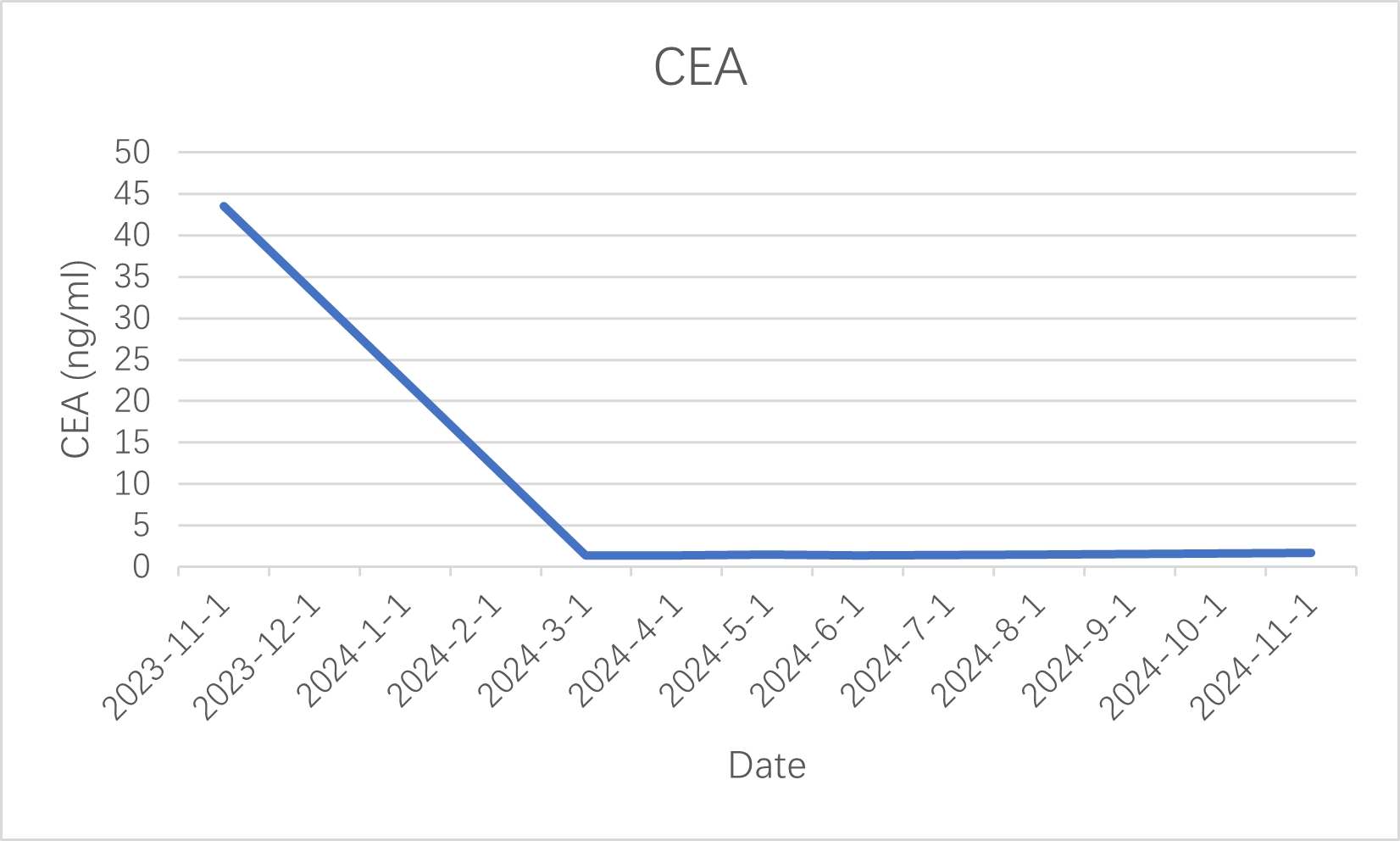

Supplement: Supplementary file 4 [file Image4.jpeg]

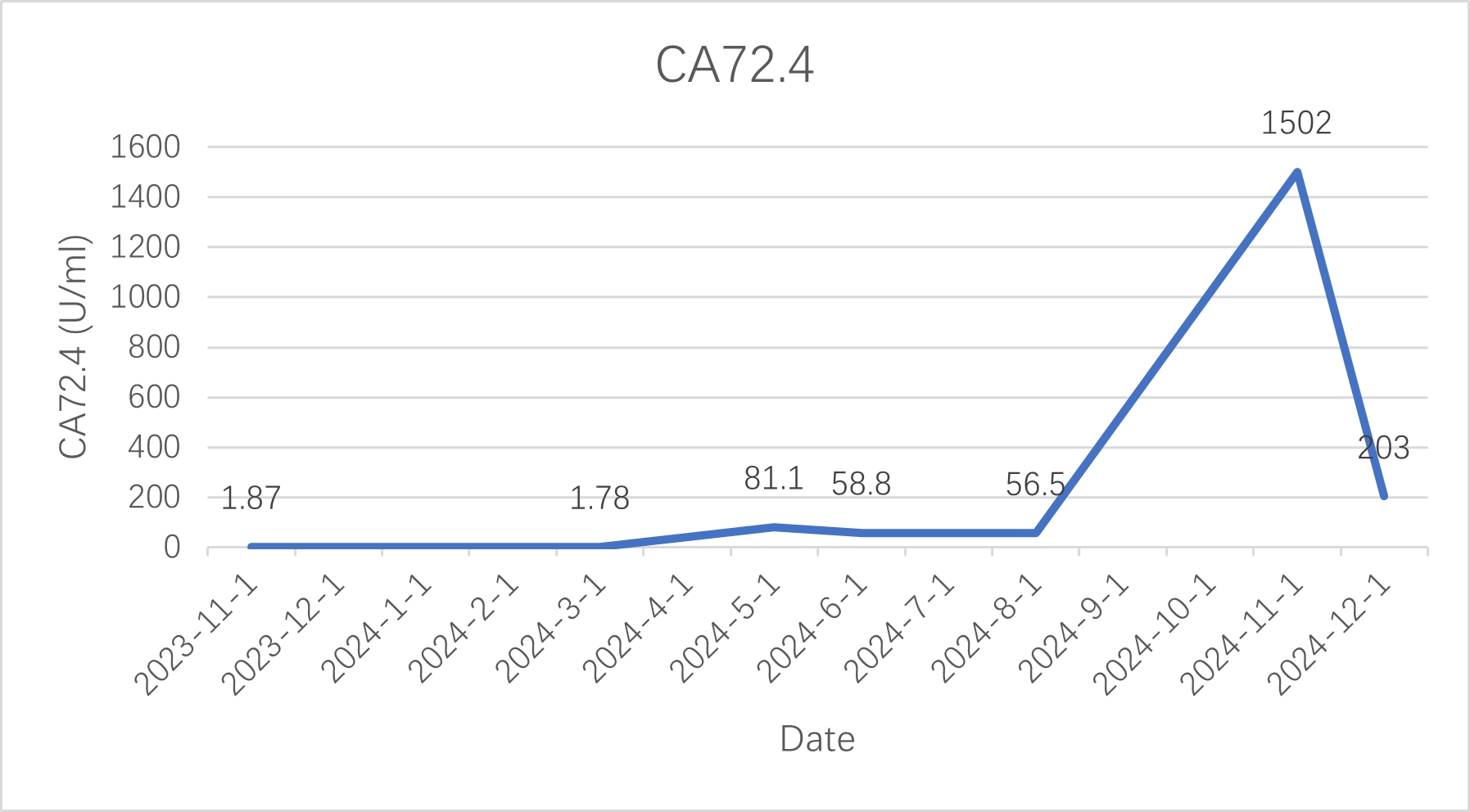

Supplement: Supplementary file 5 [file Image5.jpeg]

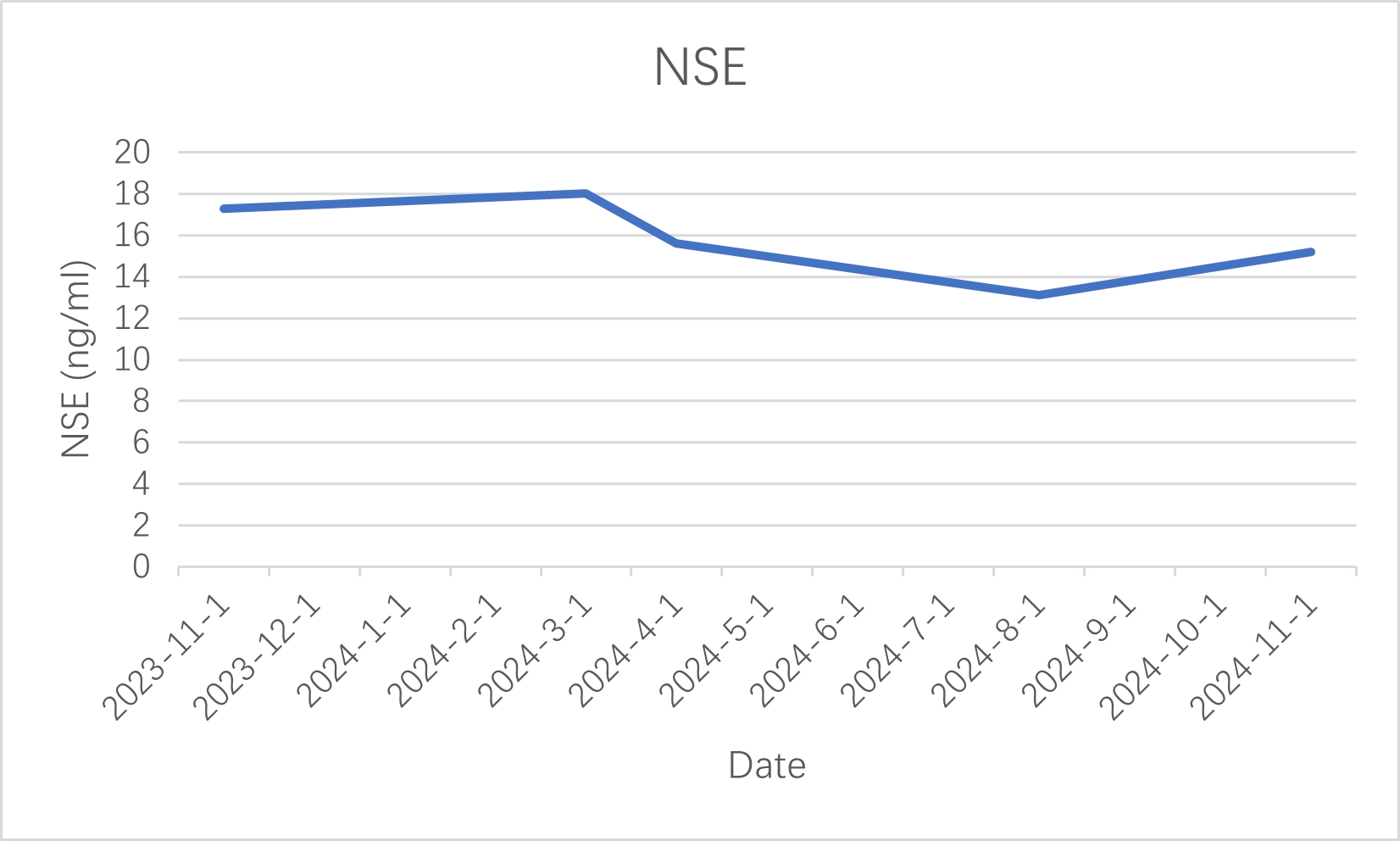

Supplement: Supplementary file 6 [file Image6.jpeg]
