# Supplementary material for: Metastatic squamous cell carcinoma of unknown primary: a case report and brief literature review
Source: Front Oncol. 2025 Oct 24;15:1613500. doi: 10.3389/fonc.2025.1613500 (PMC12591943; doi:10.3389/fonc.2025.1613500)
Supplement: Supplementary file 7 [file Table1.docx]

Supplementary Table 1. Timeline for relevant events.

| Date | Procedure | Findings |
| --- | --- | --- |
| Oct-2020 | Incidental finding | CA19-9 elevated to 56 U/ml (normal range: 0-37 U/ml) |
| Nov-2020 | Imaging studies | No abnormalities |
| May-2022 | Colonoscopy | No abnormalities detected |
| Mar-2023 | Non-contrast abdominal CT | Subphrenic mass near spleen |
| Apr-2023 | Gastroscopy + polypectomy | 2×1.5 cm pedunculated polyp near the cardia of the gastric greater curvature; pathological diagnosis: hyperplastic polyp |
| 13-Nov-2023 | Tumor markers | CEA: 43.52 ng/ml (normal range: 0-5 ng/ml); CA19-9: 387 U/ml; NSE: 17.3 ng/ml (normal range: 0-15.2 ng/ml) |
| Nov-2023 | Abdominal and pelvic CT | 5cm spindle-shaped lesion below left diaphragm (ill-defined borders) |
| 11-Dec-2023 | FDG PET-CT | Left diaphragmatic mass (SUVmax=11.4), pleural thickening (SUVmax=2.3) |
| 5-Jan-2024 | Diaphragmatic mass + splenectomy | Pathology: 6cm moderately differentiated SCC, PD-L1 CPS=40 |
| Mar-Apr 2024 | Chemotherapy (2 cycles) | Two cycles of chemotherapy (albumin-bound paclitaxel, carboplatin, pembrolizumab) |
| 26-Mar-2024 | Canhelp®-Origin test | Possible origin: cervix uteri |
| 3-Apr-2024 | Gynecological exam + colposcopy | No abnormalities detected; cervical pathology showed atrophic signs, high-risk HPV subtypes negative |
| 28-Nov-2024 | Follow-up markers + PET-CT | CA724 elevated to 1502 U/ml (normal range: 0-6.7 U/ml); no abnormalities in CT scans |
| 5-Dec-2024 | Follow-up | CA724 decreased to 203 U/ml |
| Apr-2025 | Follow-up | Disease-free survival: 15 months |
